# An Autophagy Modifier Screen Identifies Small Molecules Capable of Reducing Autophagosome Accumulation in a Model of CLN3-Mediated Neurodegeneration

**DOI:** 10.3390/cells8121531

**Published:** 2019-11-27

**Authors:** Anton Petcherski, Uma Chandrachud, Elisabeth S. Butz, Madeleine C. Klein, Wen-Ning Zhao, Surya A. Reis, Stephen J. Haggarty, Mika O. Ruonala, Susan L. Cotman

**Affiliations:** 1 Center for Genomic Medicine, Department of Neurology, Massachusetts General Hospital Research Institute, Harvard Medical School, Boston, MA 02114, USA; apetcherski@mednet.ucla.edu (A.P.); uchandrachud@mgh.harvard.edu (U.C.); ebutz1@mgh.harvard.edu (E.S.B.); mcklein@mgh.harvard.edu (M.C.K.); wnzhao@mgh.harvard.edu (W.-N.Z.); sreis@mgh.harvard.edu (S.A.R.); shaggarty@mgh.harvard.edu (S.J.H.); 2 Center for Membrane Proteomics, Goethe University of Frankfurt, 60438 Frankfurt am Main, Germany; mika@icit.bio

**Keywords:** autophagy, CLN3, Batten disease, neuronal ceroid lipofuscinosis

## Abstract

Alterations in the autophagosomal–lysosomal pathway are a major pathophysiological feature of CLN3 disease, which is the most common form of childhood-onset neurodegeneration. Accumulating autofluorescent lysosomal storage material in CLN3 disease, consisting of dolichols, lipids, biometals, and a protein that normally resides in the mitochondria, subunit c of the mitochondrial ATPase, provides evidence that autophagosomal–lysosomal turnover of cellular components is disrupted upon loss of CLN3 protein function. Using a murine neuronal cell model of the disease, which accurately mimics the major gene defect and the hallmark features of CLN3 disease, we conducted an unbiased search for modifiers of autophagy, extending previous work by further optimizing a GFP-LC3 based assay and performing a high-content screen on a library of ~2000 bioactive compounds. Here we corroborate our earlier screening results and identify expanded, independent sets of autophagy modifiers that increase or decrease the accumulation of autophagosomes in the CLN3 disease cells, highlighting several pathways of interest, including the regulation of calcium signaling, microtubule dynamics, and the mevalonate pathway. Follow-up analysis on fluspirilene, nicardipine, and verapamil, in particular, confirmed activity in reducing GFP-LC3 vesicle burden, while also demonstrating activity in normalizing lysosomal positioning and, for verapamil, in promoting storage material clearance in CLN3 disease neuronal cells. This study demonstrates the potential for cell-based screening studies to identify candidate molecules and pathways for further work to understand CLN3 disease pathogenesis and in drug development efforts.

## 1. Introduction

CLN3 disease, classified as a lysosomal disease, is the classical juvenile onset form of neuronal ceroid lipofuscinosis (NCL), or Batten disease, and it is the most common form of childhood-onset neurodegeneration. CLN3 disease is caused by mutations in the *CLN3* gene, found on chromosome 16p11.2, encoding a multipass transmembrane protein [1]. In CLN3 disease patients, vision loss between ~4 and 8 years of age is typically the first recognized symptom, followed by cognitive impairment and onset of seizures. A progressive decline in cognition and motor function is seen over the next decade of life, and late-onset cardiac symptoms can develop [2,3]. Currently, palliative care to manage symptoms is the only treatment option, and CLN3 disease is fatal, with life expectancy not typically exceeding the early twenties [2]. Despite the identification of the *CLN3* gene nearly 25 years ago [1], a thorough understanding of CLN3 protein function and disease pathogenesis is still lacking. However, a robust set of genetic disease models has been developed, in which cell biological and biochemical phenotypes have been defined [4,5]. These phenotypes largely converge on the endosomal–autophagosomal–lysosomal system, consistent with this being the primary localization of the CLN3 protein, both in neurons and non-neuronal cells [6,7].

Disruption of efficient autophagy–lysosomal flux is a common finding in lysosomal storage and neurodegenerative diseases, and it is postulated that this plays an important role in the eventual demise of neuronal cell function, since it is evident from studies of knockout models of key autophagy genes that a functioning autophagy pathway is required for neuronal health and survival [8,9]. In the case of CLN3 disease, the loss of CLN3 function has been shown to cause early-stage abnormalities in autophagy, including an accumulation of autophagosomes and autolysosomes, even preceding detectable accumulation of lysosomal storage material, and a number of studies suggest that CLN3 is required for the late stage maturation of autophagosomes/autolysosomes [10,11,12,13,14]. Given that autophagy defects are seen even in the absence of detectable lysosomal storage in CLN3 disease models, it is likely that the autophagy dysfunction is not just a consequence of storage material accumulation, but rather that it lies more upstream in the pathophysiological disease process. Taken together, these observations have led to multiple efforts to identify factors that would promote autophagy–lysosomal flux in CLN3 disease, as a possible beneficial treatment. To this end, there is a growing body of evidence in support of exploring mammalian target of rapamycin (mTOR)-independent mechanisms in CLN3 disease, which have been shown in several reports to alleviate the abnormal autophagy–lysosomal flux that is observed in the absence of CLN3 function. For example, Chang et al. reported that lithium treatment could eliminate the autophagic defects observed in Cb*Cln3^∆ex7/8/∆ex7/8^* cells and in CLN3 knock-down SH-SY5Y cells through inhibition of inositol monophosphatase (IMPase) [11]. More recently, Palmieri et al. reported that trehalose treatment of homozygous *Cln3^∆ex7/8^* mice, which accurately mimic genetic and pathological aspects of CLN3 disease [15], led to reduced lysosomal storage, reduced neuroinflammation, and improved neurobehavioral measures [16]. Trehalose was demonstrated to induce autophagy by inhibition of Akt, which caused TFEB activation in an mTOR-independent manner [16].

We previously developed and piloted a green fluorescent protein-microtubule-associated protein 1 light chain 3 (GFP-LC3) screening assay that was used in proof-of-concept studies to identify modifiers of autophagy in a murine neuronal cell model of CLN3 disease, in an unbiased fashion. Here, we have expanded on this work, further optimizing our GFP-LC3 screening assay and carrying out a larger unbiased screen of ~2000 bioactive compounds, which has highlighted a more comprehensive set of autophagy modifiers and, in particular, has now identified small molecules that reduce the accumulated autophagosomes in a model of CLN3-mediated neurodegenerative disease. Several compounds tested here in follow-up assays were also active in restoring lysosomal subcellular positioning and in clearing NCL-type lysosomal storage material. The pathways and specific drugs identified in this study corroborate and extend our earlier findings, setting the stage for further hypothesis-driven investigation of CLN3 disease pathogenesis and in future drug development efforts.

## 2. Materials and Methods

### 2.1. Maintenance of CbCln3 Cells

The establishment of Cb*Cln3*^+/+^ and Cb*Cln3^∆ex7/8/∆ex7/8^* cells stably expressing GFP-LC3 has been previously described [13,17]. The pCAG-EGFP-LC3 expression plasmid used to make the cell lines was a generous gift from Dr. Noboru Mizushima. Cb*Cln3*^+/+^ and Cb*Cln3^∆ex7/8/∆ex7/8^* GFP-LC3 cultures, or Cb*Cln3*^+/+^ and Cb*Cln3^∆ex7/8/∆ex7/8^* cultures without GFP-LC3, were maintained for this study by growing in 100 mm plastic tissue culture dishes, in Cbc culture media (Dulbecco’s modified Eagle’s medium (DMEM, high glucose, pyruvate, Thermo Fisher Scientific Inc., Pittsburgh, PA, USA), 10% heat-inactivated FBS (Sigma-Aldrich Co., St. Louis, MO, USA), 24 mM KCl, 1X penicillin/streptomycin/glutamine (Thermo Fisher Scientific Inc.), and 200 µg/mL G418 (Thermo Fisher Scientific Inc.), at 33 °C, with 5% CO_2_ atmosphere. Unless otherwise noted, the cells were maintained between 30–90% confluency, as previously described [17].

### 2.2. Compounds and Screening Library

The library used in the primary screen was a custom in-house library consisting of the following: Bioactive Lipid Library 2800v4 (Biomol GmbH, Hamburg, Germany), Fatty Acid Library 2803 (Biomol GmbH), ICCB Bioactives v2 (Biomol GmbH), Kinase Inhibitor Library 2832v2.2 (Biomol GmbH), Protease Inhibitors Library 2833v2.0 (Biomol GmbH), and the Prestwick Chemical Library 3 (Prestwick Chemical, Illkirch, France). For follow-up dose–response and validation studies, the following compounds were also obtained separately from the library: Budesonide (Santa Cruz Biotechnology, Inc., Dallas, TX, USA), fluspirilene (Sigma-Aldrich Co.), indirubin-3′-monoxime (Tocris Bioscience, Minneapolis, MN, USA), lovastatin (Selleck Chemicals, Houston, TX, USA), mestranol (Sigman-Aldrich Co.), nicardipine (Santa Cruz Biotechnology, Inc.), proadifen (SKF-525A, Santa Cruz Biotechnology, Inc.), simvastatin (Sigma-Aldrich Co.), and verapamil (Santa Cruz Biotechnology, Inc.).

### 2.3. High-Content Small Molecule Screening (HCS) and Secondary Dose–Response

Cb*Cln3*^+/+^ and Cb*Cln3^∆ex7/8/∆ex7/8^* cells stably expressing GFP-LC3 [13] were briefly grown to confluency overnight, prior to the screening experiment to increase GFP-LC3 vesicle formation. The cells were then dispensed into clear-bottomed, 384-well plates at a density of 2 × 103 cells/well using a Matrix WellMate microplate dispenser (Thermo Fisher Scientific Inc.), briefly centrifuged at 50× *g*, and allowed to attach for 24 h. The following day, compounds dissolved in DMSO, or DMSO as negative control, were transferred to 384-well plates in duplicate using a CyBi-Well vario pinning robot, which transfers a volume of ~50 nanoliters (CyBio Corp., Jena, Germany). After 23.5 h of treatment, Lysotracker^®^ Red DND-99 (LTR), used as a secondary readout marker, and Hoechst 33342 (both Thermo Fisher Scientific Inc.) were added to each well to a final concentration of 500 nM and 325 nM, respectively, using the Matrix WellMate microplate dispenser, and incubated for 30 min at 33 °C, 5% CO_2_. At the 24-h time point, the cells were fixed for 30 min at room temperature by addition of freshly prepared paraformaldehyde (PFA) to a final concentration of 3.2%, followed by 3 phosphate buffered saline (PBS, pH 7.4) washes with a Power Washer PW384 (Tecan US, Inc., Durham, NC, USA). After one additional aspiration step, PBS containing 0.1% sodium azide was added to each well, and the plates were sealed and stored at 4 °C until they were imaged.

Imaging was performed using an ImageXpress Micro high–content imaging system (Molecular Devices Inc., Sunnyvale, CA, USA) with a 10× objective. MetaXpress software (v2.0.1.28, San Jose, CA, USA, 2007) was used to acquire the images. Laser- and image-based focusing were used in the DAPI channel to obtain the correct focal plane. The exposure times were, 100 ms for the DAPI channel, 500 ms for the GFP channel, and 75 ms for the Texas Red channel. Three visual fields were imaged per well. Image processing was performed with the image analysis software, CellProfiler (v2.0.11710, Broad Institute, Cambridge, MA, USA, 2012) [18]. The processing pipeline first applied a flat field correction to the images, then recognized nuclei, GFP-LC3 vesicles, LTR vesicles, and cell outlines based on LTR cellular background signal. GFP-LC3 and LTR vesicles were assigned to their respective cells, and the cell population was then divided into cells with less than 5 or 5 or more GFP-LC3 vesicles, to arrive at a % of cells with >5 vesicles/cell (“% GFP-LC3-positive cells”). For this primary readout of % GFP-LC3-positive cells, we then determined a z-score for each compound using the following equation, z = (x_i_ − µ/2σ), where x = individual compound value, µ = mean DMSO value, and 2σ = two standard deviations from the mean. Unfocused images were excluded from the analysis by focus scores calculated through an in-house blur comparison algorithm. Briefly, images were convolved by a 5 × 5-pixel kernel in ImageJ (v1.47b, NIH, Bethesda, MD, 2012), then the convolved image was divided by the original. An overall focus score was calculated from the average division result. Since out-of-focus images are more convolved than in-focus images, and therefore more similar to their artificially convolved counterparts, high focus scores indicate lack of focus. Scores above 0.49 were found to reliably indicate out-of-focus images. Contamination with phenol red from insufficient washes was detected by measurement of red fluorescence with an EnVision plate reader (ex 543, em 620; Perkin Elmer). Typical uncontaminated samples displayed fluorescent intensities of 6000–9000 a. u., phenol red contaminated samples were found to be above 10,000 a. u. Sample wells excluded for technical reasons are indicated by “NA” in the table for all compound data in the Supplemental Information (Table S1). For our secondary readout using the Lysotracker^®^ Red DND-99 dye (Thermo Fisher Scientific, L7528), we determined relative perinuclear clustering of acidic vesicles. This was achieved by identifying Lysotracker-positive vesicle and nucleus outlines in CellProfiler and measuring the shortest distance between the vesicle and the nearest nuclear border (“lysosome-to-nucleus distance”). The average cellular lysosome-to-nucleus distance was then expressed as a fold-ratio of the DMSO control. Cb*Cln3^∆ex7/8/∆ex7/8^* cells display a significantly greater lysosome-to-nucleus distance compared to Cb*Cln3*^+/+^ cells (4.65 ± 1.3 µm vs. 3.96 ± 0.9 µm, respectively, *p* < 0.0001, unpaired Student’s *t*-test), indicating a more peripheral distribution of lysosomes, a phenotype which has been previously reported [13,17]. To assess relative toxicity of the compounds on the Cb*Cln3^∆ex7/8/∆ex7/8^* cells in the primary screen, the nuclei counts were determined for each well. A compound was interpreted as toxic if the nuclei count was below 2σ from the DMSO wells mean, and marginally toxic if the nuclei count was between 1σ and 2σ from the DMSO wells mean. The nuclei count from Cb*Cln3^∆ex7/8/∆ex7/8^* DMSO wells was 322 ± 71. Pipelines used for CellProfiler analysis will be made available on cellprofiler.org upon publication.

Compounds identified as hits were annotated using the DrugBank 5.0 and PubChem^®^ databases [19,20]. Secondary dose–response across 10 different doses was performed on a subset of hits. For secondary screening, we did not perform the confluent overnight stress on the cells that had been used just prior to the primary screen, and instead the cells were plated following growth under normal, sub-confluent growth conditions. This yielded a lower basal percentage of GFP-LC3-positive cells in both the Cb*Cln3*^+/+^ and Cb*Cln3^∆ex7/8/∆ex7/8^* cell lines (1% and 10%, respectively). Microsoft Excel (version 16) and GraphPad Prism 5 (GraphPad Software, San Diego, CA, USA) were used for graphing and statistical analyses.

### 2.4. Autophagic Flux Assay

To monitor induction of autophagy upon compound treatment, we performed an autophagic flux assay, as previously described [13]. Cb*Cln3^∆ex7/8/∆ex7/8^* cells were grown in 10-cm dishes in the presence of protease inhibitors E64 (10 µg/mL) (E3132, Sigma) and pepstatin A (100 µg/mL) (P5318, Sigma) for 16 h. Compounds (10 µM) were then added and incubated for another 24 h. Lysates from DMSO-only and compound-only treated cells (24 h) were also collected to be used as controls. Cells were then briefly washed in cold PBS (pH 7.4) and lysates were prepared in 50 mM Tris, pH 7.6, 150 mM NaCl, and 0.2% Triton X-100, plus cOmplete Mini protease inhibitors (Roche Applied Science, Basel, Switzerland) and phosphatase inhibitors (PhosSTOP, Roche Applied Science). Protein quantification on the lysates was performed (BCA assay, Thermo Fisher Scientific), and 30 µg of protein from replicate samples was loaded onto NuPAGE gels for SDS-PAGE (4–12% Bistris or 10–20% Tris-glycine). Proteins were then transferred to 0.2 µm pore size nitrocellulose for subsequent immunoblotting using anti-GFP antibody (Santa Cruz Biotechnology, sc-9996, 1:1000 dilution) and anti-mouse horseradish peroxidase-conjugated secondary (GE Healthcare, Chicago, IL, USA). Blots were subsequently developed with Western Lightning^®^ Plus-ECL enhanced chemiluminescence substrate (PerkinElmer Life Sciences, Waltham, MA, USA) and exposed to Amersham Biosciences Hyperfilm™ ECL for 3–4 exposure times. Films were developed on an X-Omat automatic processor. For densitometry, blots were digitally scanned using a Bio5000 Plus scanner (Microtek, Hsinchu, Taiwan), and quantification of bands was performed using ImageJ. Load control was β-actin (antibody from Santa Cruz Biotechnology, 1:1000 dilution). Statistical analysis of densitometry data was performed in GraphPad Prism 5, using one-way ANOVA and Tukey’s multiple comparison test for post-hoc analysis. Normalized GFP-LC3II and free GFP values (normalized to load control) from DMSO-only and protease inhibitor-only wells across all blots were combined for the statistical analysis.

### 2.5. MTT Toxicity Assay

Cb*Cln3*^+/+^ and Cb*Cln3^∆ex7/8/∆ex7/8^* cells were seeded into 96-well plates at a density of 1 × 10^4^ cells per well in 100 μL. After 24 h, DMSO, or compounds prepared in DMSO, were added to the cells (doses tested were in the range of 0.3125–20 µM for fluspirilene, 1.25–40 µM for nicardipine, and 5–100 µM for verapamil). The treatments were performed in four replicates and the DMSO concentration was kept below 0.1% at all times. After 22 h, 22 μL of thiazolyl blue tetrazolium bromide (MTT, Sigma-Aldrich) solution (5 mg/mL) were added to the cell culture media and incubated for 2 h. Subsequently, wells were briefly analyzed for the formation of formazan crystals under a light microscope equipped with a 10× objective and 100 μL of MTT solubilization solution (5 mg/mL sodium dodecylsulfate, 50% dimethylformamide in 0.05 N HCl) was added to the wells. After overnight incubation, the complete solubilization of the crystals was confirmed, and absorption at λ 540 nm was measured with a Victor X3 plate reader.

### 2.6. Lysosomal Distribution Analysis

To further assess the morphological distribution of lysosomes in Cb*Cln3*^+/+^ and Cb*Cln3^∆ex7/8/∆ex7/8^* cells, LAMP-1 immunostaining was carried out following compound (or DMSO) treatment. Cells (2.5 × 10^4^) from cultures maintained under sub-confluent growth conditions were plated onto 18 mm coverslips, and cells were allowed to attach to the coverslips overnight. Cb*Cln3*^+/+^ and Cb*Cln3^∆ex7/8/∆ex7/8^* cells used in these experiments did not have the stable expression of GFP-LC3. The indicated compounds, or DMSO, were then added, and cells were incubated for a period of 24 h prior to immunostaining. Cells were fixed for 10 min in ice-cold 50:50 methanol/acetone (v/v), or alternatively in 4% PFA for 30 min, and further processed for LAMP-1 immunostaining (LAMP-1 antibody, 1D4B, Santa Cruz Biotechnology, catalog no. sc-19992, 1:200 dilution; or LAMP-1 antibody, ab24170, Abcam, 1:500 dilution). Following fixation, cells were permeabilized using 0.05% Triton X-100 diluted in PBS, and then blocked with 5% bovine serum albumin (BSA) diluted in PBS. Detection of the 1D4B LAMP-1 primary antibody was accomplished by applying goat anti-rat AlexaFluor^®^ 568 (1:800) or, for ab24170 LAMP-1 antibody, donkey anti-rabbit AlexaFluor^®^ 488 (1:500 dilution) (both from Thermo Fisher Scientific Inc.). Immunostained coverslips were mounted onto microscope slides using ProLong™ Gold Antifade Mountant with DAPI (Thermo Fisher Scientific Inc), which was allowed to cure overnight at room temperature in the dark, and coverslips were then sealed with clear nail polish. Cells were imaged on an upright epifluorescence microscope equipped for digital capture (Zeiss), using a 40× or 63× objective. For analysis, ~50 cells per genotype and/or treatment condition were scored as either displaying a perinuclear LAMP-1 distribution pattern or a peripheral LAMP-1 distribution pattern, by a blinded observer, according to previously described procedures [21,22]. GraphPad Prism 5 was used for graphing and statistical analyses of the data (one-way ANOVA, and Tukey’s multiple comparison test for post-hoc analysis).

### 2.7. Subunit c Storage Analysis

Subconfluent, non-aged Cb*Cln3^∆ex7/8/∆ex7/8^* cells do not exhibit significant NCL-type storage material. However, storage material can be induced upon confluent aging. Therefore, to evaluate impact of compounds on clearance of storage material, confluency aged Cb*Cln3^∆ex7/8/∆ex7/8^* cells were first prepared. The induction of subunit c storage in Cb*Cln3^∆ex7/8/∆ex7/8^* cells has been previously described [17]. Briefly, Cb*Cln3^∆ex7/8/∆ex7/8^* cells (not expressing GFP-LC3) were initially plated at a density of 2 × 10^5^ cells per 100 mm plate and then incubated at 33 °C in 5% CO_2_ for 10 days. After day 10, the cells were then detached by brief trypsinization and trituration, and replated at a density of 2.5 × 10^4^ cells per 18 mm coverslip (each placed inside a well of a 12-well tissue culture plate). Cells were allowed to attach to coverslips in the incubator overnight. Compounds (or DMSO) were then added the following day for a treatment period of 24 h. Following treatment, the coverslips were processed for subunit c immunostaining, following the same procedure described above for LAMP-1 immunostaining [17]. Anti-subunit c antibody has been described previously and was used at a 1:200 dilution [23]. Secondary antibody used was donkey anti-rabbit AlexaFluor^®^ 488 (1:500 dilution; Thermo Fisher Scientific Inc.). Coverslips immunostained for subunit c were imaged on an upright epifluorescence microscope equipped for digital capture (Zeiss), using a 40× objective; ~5 images were taken per coverslip (~10–15 cells per image), which were then analyzed using the Transfluor module of MetaXpress. Subunit c positive structures were identified as structures more than 15 pixels in width and intensity of at least 20,000 grayscales above local background. Nuclei were identified as DAPI-labeled structures between 30 and 70 pixels in diameter and at least 500 grayscales above local background. GraphPad Prism 5 was used for graphing and statistical analyses of the data (one-way ANOVA, and Tukey’s multiple comparison test for post-hoc analysis).

Alternatively, aged cells were replated into 6-well dishes, and compounds were added for 24 h, prior to cell scraping and cell lysate collection. Cell lysates (prepared in buffer containing 50 mM Tris, pH 7.6, 150 mM NaCl, 0.2% Triton-X-100, and protease inhibitor cocktail) were subsequently analyzed by SDS-PAGE and immunoblotting analysis to detect total subunit c levels. Protein band intensities were analyzed in Fiji/ImageJ v1.47b [24]. GraphPad Prism 5 was used for graphing and statistical analyses of the data (one-way ANOVA, and Kruskal–Wallis Test for post-hoc analysis).

## 3. Results

### 3.1. HCS Identifies Compounds that Modulate the Vesicular GFP-LC3 Burden in CbCln3^∆ex7/8/∆ex7/8^ Cells

**Figure 1:**
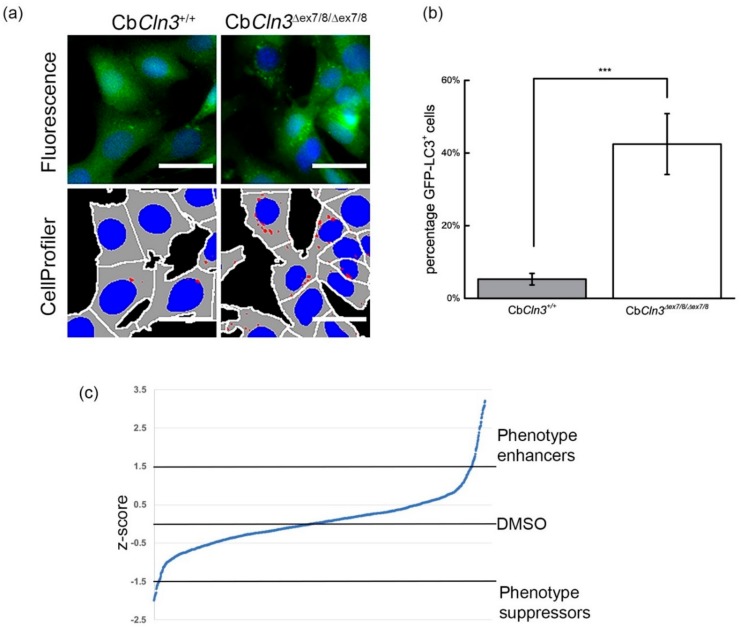
Overview of optimized GFP-LC3 high-content screening assay to monitor the abnormal accumulation of autophagosomes in Cb*Cln3^∆ex7/8∆ex7/8^* cells. (**a**) Representative images of the GFP-LC3 signal in wild-type (Cb*Cln3*^+/+^) and Cb*Cln3^∆ex7/8∆ex7/8^* cells and the masked images from CellProfiler, enabling vesicle and cellular counts in 384-well plates. In masked images, nuclei (blue), cell outline (gray), and GFP-LC3 puncta (red) are each outlined. Scale bars = 40 µm. (**b**) The relative percentages of GFP-LC3^+^ cells (defined as cells exhibiting ≥5 GFP-LC3 vesicles) in control wells (DMSO only) for wild-type (Cb*Cln3*^+/+^) and Cb*Cln3^∆ex7/8∆ex7/8^* cells are shown. Error bars represent standard deviation from the mean. *** *p* < 0.001. (**c**) Scatter plot showing the distribution of z-scores for all compounds. “Phenotype enhancers” were those compounds with a z-score of ≥1.5 (data points above line at 1.5 on graph). “Phenotype suppressors” were those compounds with a z-score of ≤−1.5 (data points below line at −1.5 on graph).

Wild-type control (Cb*Cln3*^+/+^) and Cb*Cln3^∆ex7/8/∆ex7/8^* cerebellar neuronal progenitor cells, which were derived from *Cln3*^+/+^ and *Cln3^∆ex7/8/∆ex7/8^* littermate mice and have been previously described [17], were transduced to stably express GFP-LC3 [13]. As previously reported, Cb*Cln3^∆ex7/8/∆ex7/8^* cells reproducibly display ~50% more GFP-LC3-positive vesicles under normal growth conditions, compared to control Cb*Cln3*^+/+^ cells [13]. Accumulation of LC3-positive vesicles may indicate increased autophagy induction or disruption in the endolysosomal system responsible for the degradation of autophagic cargo (reduced autophagolysosomal maturation and flux) [25]. Previous studies have suggested that the higher numbers of GFP-LC3-positive vesicles in *Cln3^∆ex7/8/∆ex7/8^* cells, also seen at the endogenous LC3 level in CLN3 patient-induced pluripotent stem cell (iPSC)-derived neurons, reflect reduced autophagolysosomal flux [10,11,13]. Therefore, in the current study, we sought to expand upon our previous work to identify additional autophagy modifying compounds, especially those that would significantly decrease the burden of GFP-LC3-positive autophagic vesicles in Cb*Cln3^∆ex7/8/∆ex7/8^* cells, reasoning that these compounds may alleviate the reduced flux seen in the absence of CLN3 function. To achieve this goal, we further optimized and expanded our previously developed autophagy screening assay [13], by establishing an optimized imaging and analysis workflow (see Materials and Methods). The assay readout in this case was the percentage of cells having 5 or more GFP-positive puncta (“% GFP-LC3-positive cells”). Using this readout, 42.5 ± 8.3% of Cb*Cln3^∆ex7/8/∆ex7/8^* cells from control wells (DMSO only) were GFP-LC3-positive, compared to 5.2 ± 1.6% of Cb*Cln3*^+/+^ cells from control wells (DMSO only) scoring as GFP-LC3-positive (Z′ = 0.19; [26]) (Figure 1a,b). Hits were identified by calculating z-scores for each compound well and applying a z-score cut-off of ≤−1.5 (“phenotype suppressors”) or ≥1.5 (“phenotype enhancers”). The full screening data for all compounds are provided in Figure 1c and Table S1 (2004 compounds in the complete library). In total, 29 unique compounds were identified as candidate phenotype suppressors (z-score ≤−1.5) (Table 1 and Table S2), while 69 unique compounds were identified as candidate phenotype enhancers (z-score ≥1.5) (Table 2 and Table S3). Notably, we also compared our new screening dataset with the dataset obtained previously in our smaller-scale screen, which utilized “mean vesicles per cell” as the assay readout, and which only identified significant hits that increased the burden of GFP-LC3 vesicles in the Cb*Cln3^∆ex7/8/∆ex7/8^* cells [13]. Two hundred and sixty-one compounds were in common across the two screens. Among the phenotype suppressors identified here, 6 compounds were also in our earlier screen, and for the phenotype enhancers, 20 compounds were also in our earlier screen. These compounds showed a significant correlation of relative activity across the two different screens, as determined by comparing the rank list for each compound present in both screens (Pearson’s correlation 0.85 for the phenotype suppressors, and 0.52, *p* < 0.05, for the phenotype enhancers; Figure S1). This was the case even though the activity of the phenotype suppressors that were identified here had not reached the z-score cut-off that had been applied in the previously reported screen [13]. Moreover, thapsigargin, which we had identified and further analyzed in our previous small-scale screen, again was among the hits that increased the GFP-LC3 burden in Cb*Cln3^∆ex7/8/∆ex7/8^* cells (Tables S1 and S3). These analyses strongly support the application of our optimized GFP-LC3 screening assay in Cb*Cln3^∆ex7/8/∆ex7/8^* cells to the broader identification of small molecules and target pathways that can modulate autophagy in the context of loss of CLN3 function, and in particular, in identifying compounds that may alleviate the abnormal accumulation of autophagosomes for further analysis as candidate drugs/drug pathways in CLN3 disease.

To gain further insight into the pathways modulating autophagy in our CLN3 disease model system, we curated predicted targets and/or mechanism of action details for the hit compounds using DrugBank and PubChem^®^ as compound-target information sources. In some cases, we also further surveyed literature describing compound activities (summarized in Tables S2 and S3). Perhaps not surprisingly, given the role of autophagy in mediating cell death, the majority of the compounds that increased the percentage of GFP-LC3-positive cells were also substantially toxic, indicated by a reduced mean nuclei count from that observed in the DMSO wells (Table S3). Interestingly, ten of these compounds are known to disrupt microtubule dynamics (gray shaded rows in Table S3). Six protease inhibitors and three topoisomerase inhibitors were also among the compounds identified as phenotype enhancers, with the latter group also showing substantial toxicity. Among the protease inhibitors, which were only marginally toxic or showed no toxicity, it was notable that three of the compounds were cathepsin B and/or L inhibitors, both enzymes that have been linked to NCL-related pathways [27,28,29]. Finally, consistent with our previous study, several calcium channel blockers were among the phenotype enhancers, including two that target endoplasmic reticulum (ER) calcium channels, like thapsigargin (Table S3, [13]).

Among the hit compounds that suppressed the accumulation of GFP-LC3 vesicles in Cb*Cln3^∆ex7/8/∆ex7/8^* cells were four calcium channel blockers (verapamil, lidoflazine, methoxy-verapamil, and nicardipine), three 3-hydroxy-3-methylglutaryl (HMG)-CoA reductase inhibitors (lovastatin, fluvastatin, and simvastatin), three estrogen receptor activators or selective estrogen receptor modulators (mestranol, estrone, and clomiphene), and two dopamine receptor inhibitors (fluspirilene and GBR 12909) (Table 1 and Table S1). Notably, fluspirilene and another hit compound, loperamide, annotated to be a dopamine receptor inhibitor and an opioid receptor inhibitor, respectively, have also been reported to show calcium channel blocking activity and to inhibit calcium flux [30,31,32,33]. 

To further interrogate our hit list, we applied a secondary screening analysis measure, incorporating Lysotracker^®^ staining to enable measurement of the average lysosome-to-nucleus distance. We have previously reported a more peripheral distribution of lysosomes in Cb*Cln3^∆ex7/8/∆ex7/8^* cells, compared to Cb*Cln3*^+/+^ cells, and we hypothesize this may contribute to the observed autophagy–lysosomal dysfunction in Cb*Cln3^∆ex7/8/∆ex7/8^* cells [10,13,17], since perinuclear lysosome positioning is important for the fusion and maturation of autophagosomes with lysosomes [21,34,35]. Across DMSO wells, Cb*Cln3^∆ex7/8/∆ex7/8^* cells averaged a lysosome-to-nucleus distance of 4.65 ± 1.3 µm, while the Cb*Cln3*^+/+^ cells averaged a lysosome-to-nucleus distance of 3.96 ± 0.9 µm, which was a 15% shorter distance than that observed in Cb*Cln3^∆ex7/8/∆ex7/8^* cells (*p* < 0.0001) (fold-difference between Cb*Cln3*^+/+^ cells and Cb*Cln3^∆ex7/8/∆ex7/8^* cells = 0.85). Interestingly, the majority of compounds that worsened the accumulation of GFP-LC3 vesicles also dramatically increased the average lysosome-to-nucleus distance in the Cb*Cln3^∆ex7/8/∆ex7/8^* cells, which was perhaps most evident for the 10 different compounds among the phenotype enhancers that disrupt microtubule dynamics (Table S3). Consistent with these observations, it is well established that destabilization of microtubules causes both a dispersion of lysosomes and an increase in LC3-positive autophagosomes that are not efficiently degraded [36,37,38,39]. Conversely, most of the phenotype suppressors had no impact on the average lysosome-to-nucleus distance in the Cb*Cln3^∆ex7/8/∆ex7/8^* cells, while five of the compounds that had reduced the accumulation of GFP-LC3 vesicles even seemed to improve the abnormal lysosomal distribution in these cells, showing a fold-difference from DMSO control of 0.85 or better, suggesting these compounds had promoted the perinuclear positioning of lysosomes (Table 1 and Table S2). Intriguingly, the five compounds that reduced the mean lysosome-to-nucleus in Cb*Cln3^∆ex7/8/∆ex7/8^* cells to wild-type levels were each connected to inhibition of calcium channels and calcium flux (Table 1 and Table S2).

Prioritizing further study of candidate phenotype suppressing compounds, follow-up dose–response analysis was carried out on a subset of the hit compounds that suppressed the GFP-LC3 burden in Cb*Cln3^∆ex7/8/∆ex7/8^* cells (Table 1). The compounds selected for testing in secondary dose–response were the following: Verapamil and nicardipine are classical calcium channel blockers and known autophagy modulators, both having been reported to induce autophagy [40,41]; fluspirilene, an antipsychotic drug, with known activity as a dopamine receptor antagonist and calcium channel blocker, is also a well-known inducer of autophagy [41]; simvastatin and lovastatin, both HMG CoA reductase inhibitors, have been linked to autophagy, with simvastatin shown to be neuroprotective and lovastatin shown to alleviate inhibition of autophagic flux by lysosomotropic agents in tumor cells [42,43,44]; ethynylestradiol-3-methyl-ether (mestranol) showed the highest activity among the three hits from the estrogen receptor class (Table 1); indirubin-3′-monoxime is an inhibitor of glycogen synthase kinase 3 beta (GSK3) and cyclin-dependent kinase 5 (CDK5), both implicated in other neurodegenerative disorders and in diseases associated with dysregulated autophagy [45,46,47]; budesonide is an anti-inflammatory glucocorticoid with established blood–brain barrier (BBB) permeability (DrugBank; https://www.drugbank.ca/drugs/DB01222); proadifen, a cytochrome P450 inhibitor, was reported to modulate the response to secondary activation of plasma membrane calcium channels [48].

In our dose–response study, in line with our primary screening data, all of the selected compounds showed a concentration-dependent reduction in the percentage of GFP-LC3-positive Cb*Cln3^∆ex7/8/∆ex7/8^* cells (Figure S2). Interestingly, a number of the compounds showed a biphasic response, promoting a reduction in the GFP-LC3-positive cells at lower doses, but an increase in GFP-LC3-positive cells at higher, toxic doses. Compounds showing this biphasic response were nicardipine, fluspirilene, simvastatin, lovastatin, indirubin-3′-monoxime, and proadifen. Verapamil, mestranol, and budesonide showed a clear dose-dependent response without any toxicity evident at the doses tested.

### 3.2. Validation of Autophagy–Lysosomal Phenotype Correction in CbCln3^∆ex7/8/∆ex7/8^ Cells by Calcium Channel Targeting Compounds

**Figure 2:**
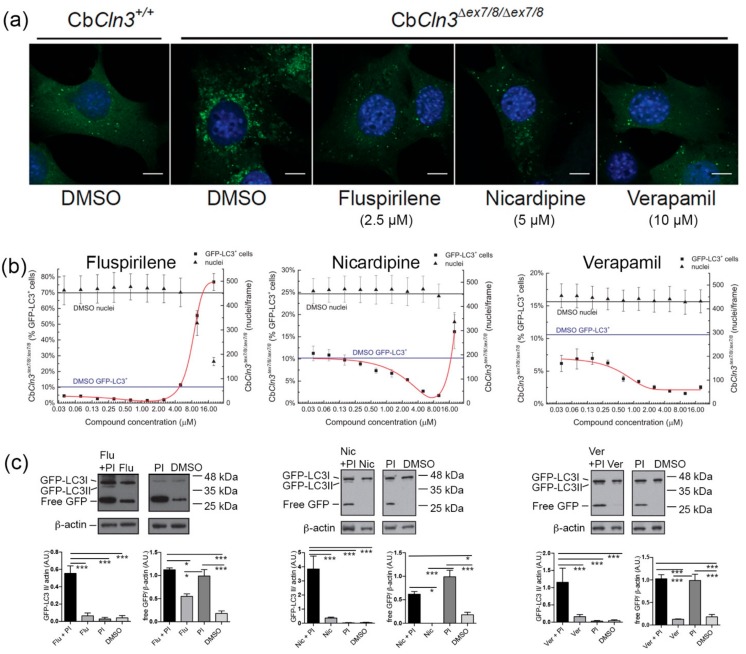
Validation of effect on GFP-LC3-positive vesicle burden for selected hit compounds. (**a**) Representative confocal microscopy images of the GFP-LC3 signal (green) in wild-type (Cb*Cln3*^+/+^) and Cb*Cln3^∆ex7/8∆ex7/8^* cells, treated for 24 h with fluspirilene (2.5 µM), nicardipine (5 µM), or verapamil (10 µM) (or DMSO as negative control) are shown. Scale bars = 10 µm. DAPI was used to label nuclei (blue). (**b**) Graphs of dose–response effect on percentage of GFP-LC3-positive cells (squares; left *y*-axis) and on nuclei count (triangles; right *y*-axis) in Cb*Cln3^∆ex7/8∆ex7/8^* cells are shown. Compound concentrations (µM) are shown on the *x*-axis. For reference, the mean values for percentage of GFP-LC3-positive cells and nuclei count for DMSO-treated wells are indicated by the solid lines (blue line = DMSO % GFP-LC3^+^ cells; black line = DMSO nuclei count). Error bars represent standard deviation from the mean (*n* = 3 experiments, each with quadruplicate wells per dose). (**c**) Representative blots of Cb*Cln3^∆ex7/8∆ex7/8^* cellular lysates are shown, probed with anti-GFP antibody, to monitor autophagic flux upon compound treatments. The GFP-LC3II, GFP-LC3I, and free GFP isoforms are indicated. Molecular weights are indicated on the right of each blot (kDa = kilodalton). The full set of fluspirilene, nicardipine, or verapamil samples, treated with compound alone, or compound plus protease inhibitors (PI) (and controls of DMSO only and protease inhibitors only), were each run on single gels/blots, but intervening replicate wells were cropped out for the figure (full blots with replicate wells are provided in Figure S3). For each compound, densitometric quantification of replicate wells is shown in the bar graphs, for GFP-LC3II levels, normalized to load control (β-actin), and for free GFP levels, normalized to load control (β-actin). In each case, the addition of compound in the presence of protease inhibitors led to a significant increase in GFP-LC3II levels, consistent with an induction of autophagic flux upon compound treatment. Free GFP levels significantly increased in the presence of protease inhibitors, as compared to DMSO control or compound-only. Treatment with compound plus protease inhibitors did not further alter free GFP levels, as expected. Significance in Tukey’s multiple comparison post-hoc test, following one-way ANOVA, is shown (* *p* < 0.05; *** *p* < 0.001).

Further validation studies were carried out on three selected compounds, which were verapamil, fluspirilene, and nicardipine. Verapamil, an anti-hypertensive agent, consistently emerged among the compounds with the most activity in suppressing the abnormal accumulation of GFP-LC3 vesicles in Cb*Cln3^∆ex7/8/∆ex7/8^* cells (Table 1 and Table S2, this study, and in [13]), and verapamil reduced the lysosome-to-nucleus distance to wild-type levels in our secondary readout (Table 1 and Table S2). It was notable that the related compound, methoxyverapamil, also consistently showed the same activities (Table 1 and Table S2). Verapamil has been shown to induce autophagic clearance and to be neuroprotective/cytoprotective in several animal models of other diseases, including Huntington’s disease and metabolic disease [49,50]. Fluspirilene is a typical antipsychotic drug, a known dopamine receptor antagonist and calcium channel blocker, and a well-known inducer of autophagy [41,49], and similar to verapamil, fluspirilene was one of the compounds that also improved the lysosome-to-nucleus phenotype in our secondary readout (Table 1 and Table S2). Nicardipine, another anti-hypertensive agent, consistently had one of the highest levels of activity in suppressing the abnormal accumulation of GFP-LC3 vesicles in Cb*Cln3^∆ex7/8/∆ex7/8^* cells (Table 1 and Table S2, this study, and in [13]), and it was reported to potentially have a preventative effect in Alzheimer’s disease through its effects on the cerebral vasculature [51]. Nicardipine has been demonstrated to have potential as a neuroprotective and anti-inflammatory agent in other neurodegenerative diseases [51,52]. Representative micrograph images of the effects of fluspirilene, nicardipine, and verapamil, and dose–response activity in the GFP-LC3 assay are shown in Figure 2a,b. We also confirmed that these compounds indeed induced autophagic flux in the Cb*Cln3^∆ex7/8/∆ex7/8^* cells, since compound treatment significantly increased GFP-LC3II levels in the presence of protease inhibitors (Figure 2c).

**Figure 3:**
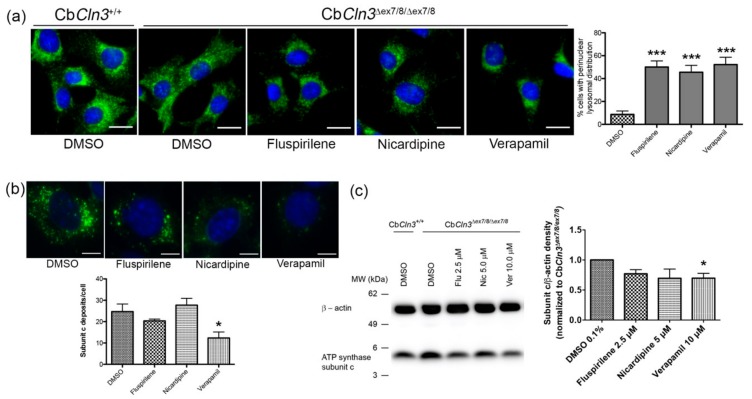
Analysis of fluspirilene, nicardipine, and verapamil effects on lysosomes and NCL-type storage material clearance. (**a**) Representative micrograph images of LAMP-1 immunostained wild-type (Cb*Cln3*^+/+^) and Cb*Cln3^∆ex7/8∆ex7/8^* cells following 24-h treatment with fluspirilene (2.5 µM), nicardipine (5 µM), or verapamil (10 µM) (or DMSO as negative control) are shown. Scale bars = 20 µm. Note the altered LAMP-1 vesicle pattern in the DMSO-treated Cb*Cln3^∆ex7/8∆ex7/8^* cells, which is shifted toward the periphery of the cells, as compared to that observed in the DMSO-treated Cb*Cln3*^+/+^ cells. Twenty-four hour treatment of Cb*Cln3^∆ex7/8∆ex7/8^* cells with fluspirilene, nicardipine, or verapamil shifted the LAMP-1 staining pattern to a more perinuclear distribution. The bar graph represents the percentage of cells with a perinuclear lysosomal distribution pattern of Cb*Cln3^∆ex7/8∆ex7/8^* cells for each treatment condition. Error bars represent standard deviation from the mean (*n* = ~20 images, from four independent experiments). *** *p* < 0.001 post-hoc analysis following one-way ANOVA. (**b**) Representative micrograph images of subunit c immunostained Cb*Cln3^∆ex7/8∆ex7/8^* cells, following 10-day aging and a subsequent 24-h treatment with the indicated compounds (10 µM, or DMSO). Bar graph represents results of image analysis for mean subunit c deposits/cell for each treatment condition, for a representative experiment (*n* = 5–7 representative image means). * *p* < 0.05 post-hoc analysis following one-way ANOVA. (**c**) A representative immunoblot is shown, probed for subunit c levels in total lysates from wild-type (Cb*Cln3*^+/+^) and Cb*Cln3^∆ex7/8∆ex7/8^* cells, which were aged for 10 days and subsequently treated for 24 h with the indicated compounds (or DMSO). An antibody recognizing β-actin was used as load control and for normalization purposes. Bar graph represents relative densitometric quantification of subunit c levels (normalized to β-actin). Data for each condition were normalized to that obtained for the DMSO-treated Cb*Cln3^∆ex7/8∆ex7/8^* cells, which was set to a value of 1. Error bars represent SEM (*n* = 5). * *p* < 0.05 post-hoc analysis following one-way ANOVA.

We next wanted to follow-up on the possibility that these compounds may also modify other lysosomal phenotypes observed in Cb*Cln3^∆ex7/8/∆ex7/8^* cells, including lysosomal positioning and the accumulation of NCL-type storage material. From our primary screen, we found that fluspirilene, nicardipine, and verapamil reduced the lysosome-to-nucleus distance in Cb*Cln3^∆ex7/8/∆ex7/8^* cells from that observed in DMSO wells, which was 4.65 ± 1.3 µm, to 3.85 ± 0.6 µm, 4.18 ± 0.3 µm, and 3.76 ± 0.5 µm, respectively, suggesting these compounds may restore lysosomal positioning in Cb*Cln3^∆ex7/8/∆ex7/8^* cells to a more perinuclear distribution like that observed in wild-type cells (Figure S4). To further assess lysosomal morphology and distribution, Cb*Cln3^∆ex7/8/∆ex7/8^* cells were incubated with fluspirilene, nicardipine, or verapamil (or DMSO-only as negative control) at subtoxic doses selected from our dose–response analysis of GFP-LC3 and nuclei count, which was independently validated in a toxicity assay (Figure S5), and lysosomes were visualized by LAMP-1 immunostaining. As shown in Figure 3a, consistent with our primary screening data, all three of these compounds significantly improved the abnormal lysosomal distribution that is observed in the Cb*Cln3^∆ex7/8/∆ex7/8^* cells, which is observed as a peripheral distribution compared to the perinuclear lysosomal distribution observed in the Cb*Cln3*^+/+^ cells when incubated with DMSO. Upon treatment with fluspirilene, nicardipine, or verapamil, ~50% of the cultured Cb*Cln3^∆ex7/8/∆ex7/8^* cells showed the wild-type-like perinuclear distribution pattern. 

Finally, we evaluated the impact of the three compounds on accumulation of subunit c of the mitochondrial ATPase, which is the main component of the lysosomal storage material in CLN3 disease [53,54] and which we have previously shown progressively accumulates in Cb*Cln3^∆ex7/8/∆ex7/8^* cells upon aging [17]. Following 10-day aging of Cb*Cln3^∆ex7/8/∆ex7/8^* cells at a confluent plating density, the cells were treated for 24 h with fluspirilene, nicardipine, or verapamil (or DMSO as negative control), prior to fixation and subunit c immunostaining, or to lysate preparation and immunoblot analysis. As shown in Figure 3b,c, a reduction in the number of subunit c deposits per cell was observed following verapamil treatment, while fluspirilene and nicardipine did not show the same degree of reduction in this measure. By immunoblot analysis, again verapamil stimulated a significant reduction in the total subunit c levels from that observed in DMSO control Cb*Cln3^∆ex7/8/∆ex7/8^* cultures (Figure 3c), while fluspirilene and nicardipine treatment did not lead to a significant reduction in subunit c levels.

## 4. Discussion

Here, we have extended our previous work [13], identifying an expanded set of autophagy modifiers in the Cb*Cln3^∆ex7/8^* neuronal cell model of CLN3 disease, including both phenotype enhancers and phenotype suppressors. Three of the top phenotype suppressors identified here were studied in further detail; fluspirilene, nicardipine, and verapamil were validated to induce autophagic flux and were shown to have activity in suppressing abnormal lysosomal positioning. In addition, verapamil was shown to also induce clearance of accumulated subunit c of the mitochondrial ATP synthase aggregates, which is the main protein in the lysosomal storage material in CLN3 disease. Upon examination of the full list of hit compounds, several target pathways were highlighted that will be of interest for future studies to better understand their possible role in the disruption of autophagy and other pathogenic mechanisms in CLN3 disease. These include the regulation of calcium flux, microtubule dynamics, and the mevalonate pathway.

The identification of a number of compounds targeting calcium channels and calcium flux that could modulate CLN3 disease-related phenotypes in the current study was consistent with our previous work and that of others, where it has been shown that intracellular calcium handling, depolarization-dependent calcium influx, and calcium-induced cytotoxicity are altered in CLN3 deficient cells [13,55,56]. In a previous study, we identified a sensitivity of CLN3-deficient cells to the autophagic response to thapsigargin, a SERCA inhibitor, which was mediated via alterations in intracellular calcium handling [13]. While ER calcium stores were not themselves altered, other subcellular calcium pools showed altered calcium levels in the Cb*Cln3^∆ex7/8/∆ex7/8^* cells, including mitochondria and lysosomes [13]. Several calcium channel blockers have also previously been reported to impact other phenotypes in CLN3 disease models. For example, five different calcium channel blockers were reported to have a partial effect on lengthening lifespan in a triple *cln3* gene knockout *Caenorhabditis elegans* model and on preventing etoposide-induced apoptosis in CLN3 knockdown primary rat neurons [57,58]. However, out of these five compounds, only one also had activity in our current study (nicardipine), while the other four were inactive in our primary screening analysis. These discrepancies may be due to the differences in the assays and cellular systems across the different studies. Our finding that calcium-modulating compounds influence autophagy–lysosomal flux is in strong agreement with other studies on autophagy; among other calcium channel blockers, verapamil and fluspirilene in particular, have each been shown in other systems to enhance autophagic flux in a calcium-dependent fashion, leading to autophagic clearance and, in the case of verapamil, protection from neurodegeneration [33,41,49,59]. Taken together, it is likely that alterations in calcium play a role in the autophagy–lysosomal defects observed in CLN3 disease. 

The calcium-related compounds identified in the current study also establish several important new tools and candidate leads for further drug development efforts. In particular, verapamil consistently showed beneficial effects on autophagy–lysosomal phenotypes, suggesting that further study of this drug in preclinical models and assays of CLN3 disease is warranted. Notably, verapamil was previously reported to have beneficial effect in a mouse model of obesity-related metabolic disease, in which there is reduced autophagy–lysosomal flux due to the calcium response to hepatic lipotoxicity [50]. It is also noteworthy that, like in the other studies on enhanced autophagic flux in models of CLN3 disease, the validated hit compounds identified here are also thought to mediate the promotion of autophagy through mTOR-independent mechanisms, involving IP_3_ levels and the regulation of calpain activity [49,59]. However, genetic modifiers of phenotypes in a CLN3 disease yeast model have been reported to strongly converge on Tor signaling, which the authors suggest may be dysfunctional in the absence of the CLN3 protein [60]. Given the important role of the lysosome in regulating mTOR signaling [61,62,63], further studies are warranted to fully delineate the role of mTOR and other pathways affected by loss of CLN3 function and how targeting these pathways will impact CLN3 disease. It may be that targeting multiple pathways will ultimately be needed to achieve the greatest success in compensating for the effects of CLN3 deficiency. In addition to the pathways already discussed herein, stress–response, valine catabolism, and ROCK2 signaling pathways have also been implicated in possibly modulating the CLN3 disease process [17,64,65,66,67,68,69].

The observation that a number of compounds well known to disrupt microtubule dynamics substantially worsened the GFP-LC3 autophagosome burden was also an intriguing outcome of our study, suggesting another possible mechanism by which autophagy–lysosome flux is reduced upon loss of CLN3 protein function, as these compounds also worsened the peripheral distribution of lysosomes that is observed in the Cb*Cln3^∆ex7/8/∆ex7/8^* cells. Disruption of the actin cytoskeleton upon CLN3 deficiency has been reported, and altered ARF1–Cdc42 signaling, which regulates actin assembly/disassembly, was demonstrated in murine cells lacking CLN3 [70,71,72]. CLN3 has also been reported to interact with actin-associated proteins, including fodrin [71], myosin-IIb [73], and Hook1 [74], supporting a possible connection between CLN3 function and actin cytoskeleton regulation that could impact autophagy and other vesicular trafficking pathways, such as endocytosis [72].

Finally, it was intriguing that three statins were identified as candidate phenotype suppressors in our autophagy screen. Statins are inhibitors of HMG CoA reductase, the rate limiting enzyme in the mevalonate pathway responsible for the biosynthesis of isoprenoids like cholesterol. In addition to lowering cholesterol, complex additional biological activities are being uncovered for the statins, including in the regulation of oxidative stress, apoptosis, and autophagy [42,75]. Further study of the role of the mevalonate pathway in CLN3 disease is needed to shed light on the significance of these findings and the potential for statins in the treatment of CLN3 disease. Indeed, these studies are already in progress (Ruonala et al., manuscript in preparation [76]). 

In summary, we have established an optimized high-content screening assay in an accurate genetic model of CLN3 disease that has been demonstrated to successfully identify small molecule modifiers of autophagy and target pathways for further study to advance our understanding of CLN3 disease pathophysiology. The identification of a set of compounds that robustly corrected autophagy–lysosomal phenotypes in our CLN3 disease model suggests new candidate pathways for future research and drug development efforts.

## Figures and Tables

**Table 1:** Phenotype suppressors from primary high-content small molecule screening (HCS).

Compound Name	Mean z-Score Cb*Cln3^∆ex7/8/∆ex7/8^*	% GFP-LC3-Positive Cb*Cln3^∆ex7/8/∆ex7/8^* Cells (DMSO = 42.5%) [Value from Duplicate Well, if Compound Appeared Twice in Hit List]	Tested in Dose–Response Secondary Analysis	Fold Change to Lysosome-to-Nucleus Distance, Compared to DMSO (for Reference, DMSO Wild-Type Cells: DMSO Cb*Cln3^∆ex7/8/∆ex7/8^* Cells Ratio = 0.85)
lovastatin *	−2	9 [11.6]	Yes	0.91
mestranol (ethynylestradiol 3-methyl ether)	−1.99	9.2	Yes	0.87
thonzonium bromide	−1.97	9.5	No	0.86
20-Carboxy-leukotriene B4	−1.93	10.2	No	1.02
estrone	−1.9	10.8	No	0.98
verapamil	−1.89	10.9	Yes	***0.8***
fluspirilene *	−1.89	11 [16.2]	Yes	***0.81***
phenamil	−1.86	11.4	No	1.01
lidoflazine	−1.85	11.6	No	***0.85***
forskolin	−1.83	12	No	1.06
methoxy-verapamil	−1.82	12	No	***0.84***
indirubin-3′-oxime	−1.82	12.1	Yes	1.08
fluvastatin	−1.81	12.3	No	0.96
9(E)-Hexadecenoic acid	−1.71	13.9	No	0.92
clomiphene	−1.68	14.5	No	1
nicardipine	−1.67	14.6	Yes	0.9
methyl benzethonium chloride	−1.64	15.2	No	1.19
benzethonium chloride	−1.63	15.3	No	1.07
ethaverine	−1.62	15.5	No	1.1
proadifen	−1.61	15.5	Yes	0.94
simvastatin	−1.61	15.6	Yes	1.01
clofazimine	−1.59	16	No	0.94
butylparaben	−1.54	16.7	No	0.95
PP1	−1.54	16.8	No	1.05
budesonide	−1.54	16.8	Yes	1.14
GBR 12909	−1.54	16.8	No	0.9
deptropine	−1.54	16.8	No	0.96
loperamide	−1.52	17.1	No	***0.77***
damnacanthal	−1.51	17.3	No	1.13

* designates compounds that appeared twice in the hit list, from two independent wells. Bold and italic numbers indicate compounds that induced a fold change in lysosome-to-nucleus distance to wild-type levels.

**Table 2:** Phenotype enhancers from primary HCS screen.

Compound Name	Mean z-Score Cb*Cln3^∆ex7/8/∆ex7/8^*	% GFP-LC3-Positive Cb*Cln3^∆ex7/8/∆ex7/8^* cells (DMSO = 42.5%) [Value from Duplicate Well, if Compound Appeared Twice in Hit List]	Fold Change to Lysosome-to-Nucleus Distance, Compared to DMSO (for Reference, DMSO Wild-Type Cells: DMSO Cb*Cln3^∆ex7/8/∆ex7/8^* Cells Ratio = 0.85)
Ro 31-8220 *	3.19 [1.95]	95.8 [75]	3.96 [1.27]
merbromin	3.17	95.4	0.88
ellipticine	3.17	95.4	1.44
TPEN	3.16	95.2	2.2
MG-132	3.08	93.9	2.6
puromycin *	3.07 [2.14]	93.7 [78.2]	3.24 [1.38]
FCCP	3.06	93.6	1.28
prazocin	3.03	93.1	1.23
Ac-Leu-Leu-Nle-CHO	3.03	93	3.11
chelidonine (+)	3.03	93	3.41
parbendazole	3.01	92.7	6.75
bafilomycin A1	2.99	92.4	0.77
erbstatin analog	2.99	92.4	2.73
ikarugamycin	2.97	92.1	2.48
manumycin A	2.95	91.7	2.09
nocodazole	2.94	91.6	3.77
colchicine	2.88	90.6	4.26
piperlongumine	2.85	90.1	3.43
mebendazole	2.85	90	4.29
albendazole	2.84	89.9	3.34
taxol (paclitaxel) *	2.78 [2.5]	89 [84.3]	2.04 [5.16]
podophyllotoxin	2.75	88.4	3.9
chrysene-1,4-quinone	2.75	88.3	3.46
tyrphostin 9 *	2.74 [1.75]	88.2 [71.8]	1.23 [1.23]
quinacrine	2.64	86.6	1.14
penitrem A	2.64	86.6	1.59
azacytidine-5	2.61	86.1	1.07
BAPTA-AM	2.55	85.1	1.19
arvanil	2.53	84.8	1.39
vinblastine	2.53	84.8	6.44
thapsigargin	2.53	84.7	2.78
azaguanine-8	2.52	84.5	1.31
latrunculin B	2.49	84	3.26
methiazole	2.46	83.6	3.05
E6 berbamine	2.45	83.4	0.84
scoulerine	2.37	82.1	2.28
Ala-Ala-Phe-CMK (AAF-CMK) *	2.36 [1.9]	81.8 [74.2]	1.05 [0.89]
ciclopirox ethanolamine	2.3	80.9	1.45
triciribine	2.29	80.6	2.68
actinomycin D	2.28	80.5	1.56
maprotiline	2.24	79.9	0.84
Hoechst 33342	2.19	79	2.66
TLCK	2.17	78.8	1.11
fumagillin	2.16	78.5	0.99
triptolide	2.13	78.1	2.17
disulfiram	2.08	77.6	2.21
AG-879 *	2.05 [1.97]	76.7 [75.4]	2.46 [1.64]
SKF-96365	2.01	76	3.23
menadione	2	75.9	1.08
shikonin	1.98	75.5	0.96
cytochalasin D	1.96	75.2	2.44
curcumin	1.95	75	0.92
tetrandrine *	1.93 [1.52]	74.6 [67.9]	0.77 [0.85]
camptothecine (S,+)	1.81	72.8	4.93
hycanthone	1.81	72.8	1.15
geldanamycin	1.75	71.6	2.56
5-iodotubercidin	1.74	71.5	1.24
wiskostatin	1.72	71.1	0.84
trichostatin-A	1.7	70.8	1.31
Tosyl-Phe-CMK (TPCK)	1.69	70.8	2.75
Z-FA-FMK	1.69	70.7	0.94
DRB (NSC 401575)	1.65	70.1	1.18
dilazep	1.64	69.9	1.01
6-formylindolo [3,2-B] carbazole	1.64	69.8	0.96
CA-074 Me *	1.63 [1.62]	69.8 [69.5]	0.96 [0.9]
2,5-ditertbutylhydroquinone	1.63	69.6	1.24
NapSul-Ile-Trp-CHO *	1.6 [1.59]	69.2 [69]	1.41 [0.9]
raloxifene	1.57	68.8	0.79
piceatannol	1.55	68.3	0.92

* designates compounds that appeared twice in the hit list, from two independent wells.

## Supplementary Materials

The following are available online at https://www.mdpi.com/2073-4409/8/12/1531/s1: Figure S1. Relative activity comparison for 261 compounds that were common in the current screen and in a smaller-scale screen reported in 2015; Figure S2. Dose–response of selected hit compounds; Figure S3. Anti-GFP immunoblots to monitor autophagic flux; Figure S4. Average lysosome-to-nucleus measurements from primary screen; Figure S5. MTT assay toxicity analysis for fluspirilene, nicardipine, and verapamil; Table S1. All compounds’ screening data; Table S2. Phenotype suppressors that reduced GFP-LC3 autophagosome burden; Table S3. Phenotype enhancers that increased GFP-LC3 autophagosome burden.
