# Supplementary material for: An Autophagy Modifier Screen Identifies Small Molecules Capable of Reducing Autophagosome Accumulation in a Model of CLN3-Mediated Neurodegeneration
Source: Cells. 2019 Nov 27;8(12):1531. doi: 10.3390/cells8121531 (PMC6953052; doi:10.3390/cells8121531)
Supplement: Supplementary file 1 [file cells-08-01531-s001.zip › Supplementary Materials Rev2/Petcherski_SupplementalFigures_Rev2.pdf]

Figure S1.

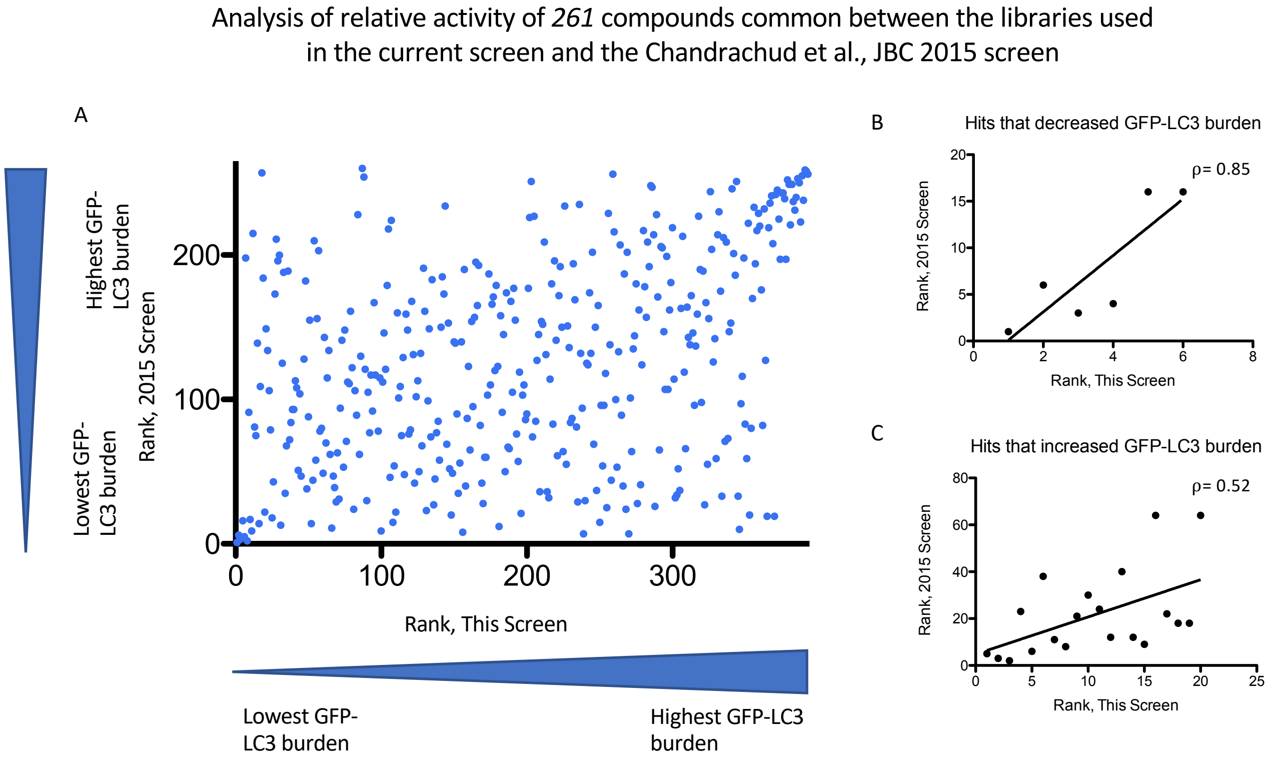

Figure S1. Relative activity comparison for 261 compounds that were common in the current screen and in a smaller-scale screen reported in 2015. A rank list of relative activity (from z-scores, representing the lowest to highest GFP-LC3 burden) was prepared for the compounds in common for each of the screens. Assay readouts from the two different screens were % GFP-LC3-positive cells (this screen) or mean vesicle count per cell (2015 screen. Chandrachud et al., J Biol Chem, 2015), which were then converted to z-scores (see Methods and Chandrachud et al., J Biol Chem, 2015). A) An XY scatter plot for rank values of all compounds is shown (this screen, x-axis; 2015 screen, y-axis). B) A scatter plot is shown for only the hit compounds that decreased GFP-LC3 burden that were identified in the current screen, but which had also been present in the 2015 screen. The Pearson's correlation (ρ) was determined (= 0.85), which was significant in a two-tailed analysis ($P<0.05$). C) A reverse rank list was generated for better visualization of correlation among the hits that increased the GFP-LC3 burden, and the scatter plot in C shows this reverse ranks for only the hit compounds identified in the current screen, but which had also been present in the 2015 screen. The Pearson's correlation (ρ) was determined (= 0.52), which was significant in a two-tailed analysis ($P<0.05$).

Chandrachud, U.; Walker, M.W.; Simas, A.M.; Heetveld, S.; Petcherski, A.; Klein, M.; Oh, H.; Wolf, P.; Zhao, W.N.; Norton, S., et al. Unbiased Cell-based Screening in a Neuronal Cell Model of Batten Disease Highlights an Interaction between Ca2+ Homeostasis, Autophagy, and CLN3 Protein Function. *J Biol Chem* **2015**, *290*, 14361-14380, doi:10.1074/jbc.M114.621706.

Figure S2.

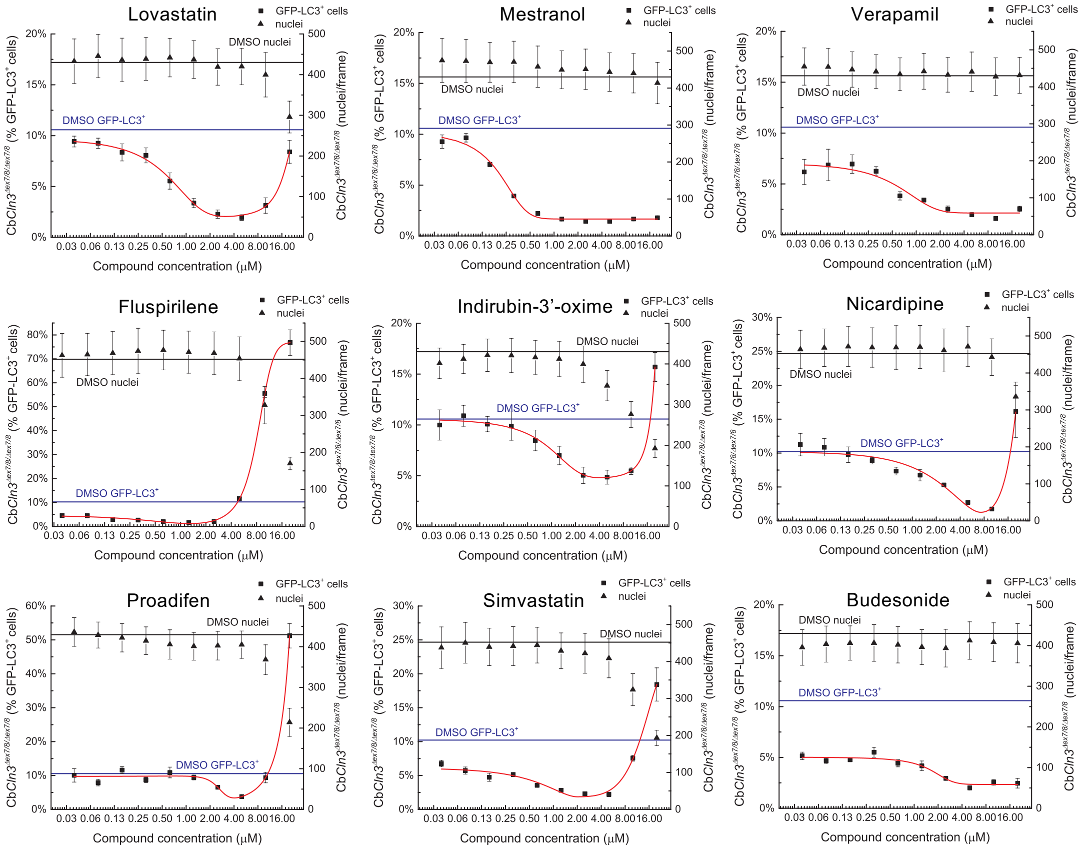

Figure S2. Dose-response of selected hit compounds. Graphs of dose-response effect on percentage of GFP-LC3-positive cells (squares; left y-axis) and on nuclei count (triangles; right y-axis) in Cb*Cln3*$^{\Delta ex7/8\Delta ex7/8}$ cells are shown for all of the compounds that were selected for validation in dose-response experiments. Compound concentrations (μM) are shown on the x-axis. For reference, the mean values for percentage of GFP-LC3-positive cells and nuclei count for DMSO-treated wells are indicated by the solid lines (blue line= DMSO % GFP-LC3+ cells; black line= DMSO nuclei count). Error bars represent standard deviation from the mean, determined from three separate experiments (n=4 replicates in each experiment).

Figure S3.

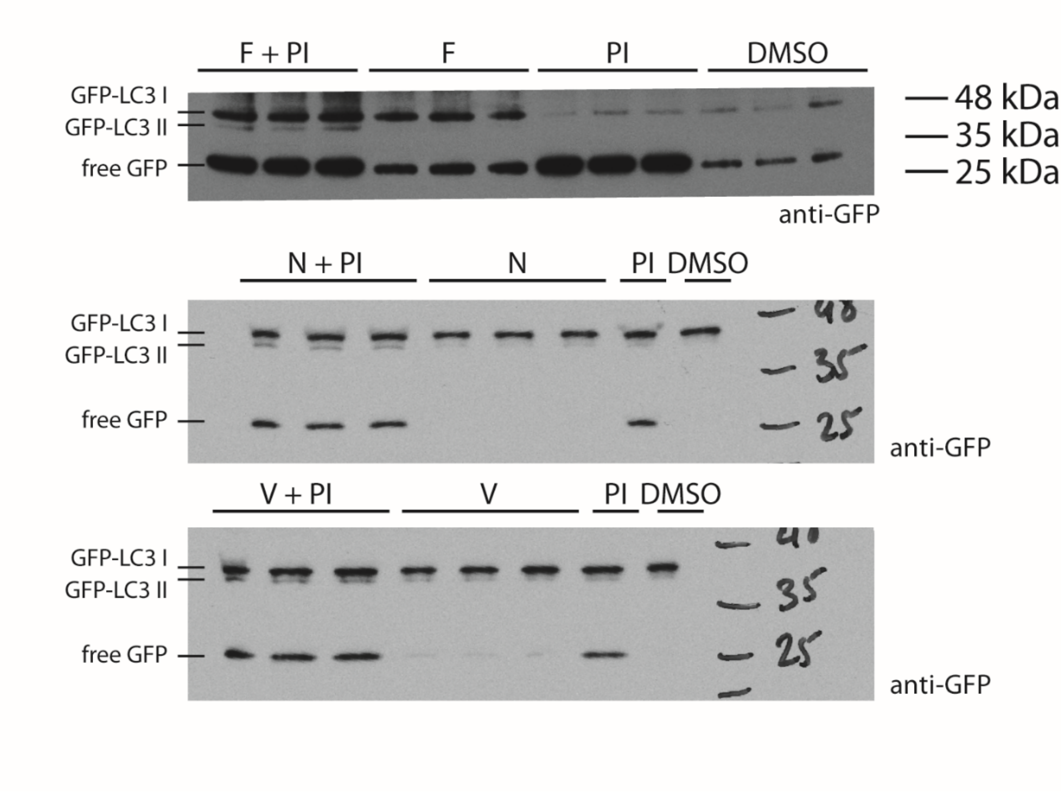

Figure S3. Anti-GFP immunoblots to monitor autophagic flux. Immunoblots showing triplicate lysates from stable expressing GFP-LC3 Cb*Cln3*$^{\Delta ex7/8/\Delta ex7/8}$ cells treated for 24h with Fluspirilene (F), Nicardipine (N) and Verapamil (V) (10 µM), either alone or in combination with protease inhibitor (PI) (see Methods for full experimental details). Lysates from PI and DMSO treated cells were used as controls. Molecular weights are indicated on the right (kDa= kilodalton). The different isoforms of the transgene GFP-LC3 (GFP-LC3I, GFP-LC3II, and free GFP) are indicated to the left of the blots.

Figure S4.

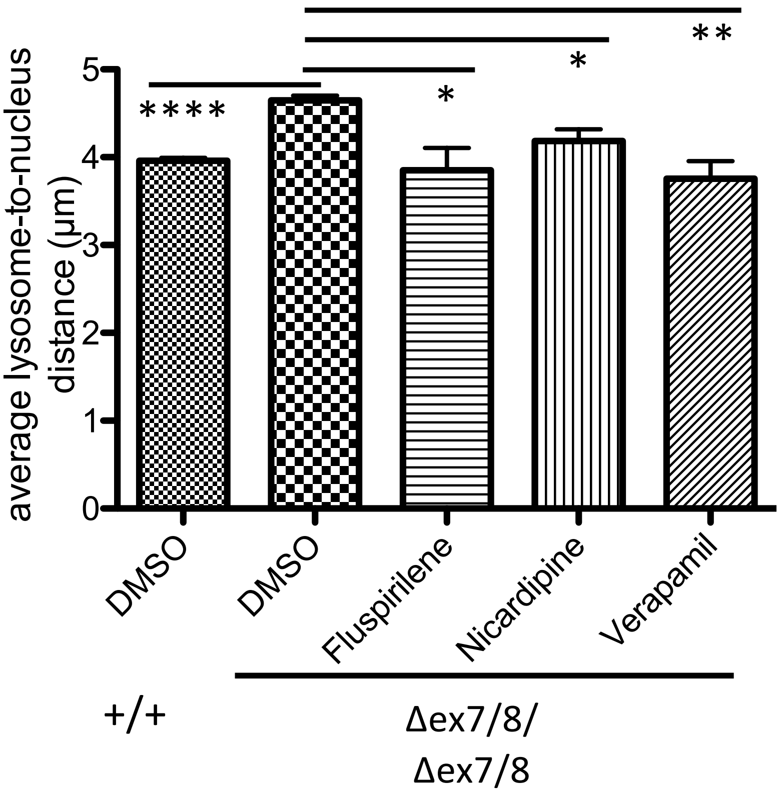

Figure S4. Average lysosome-to-nucleus measurements from primary screen. Bar graph shows the average lysosome-to-nucleus distance determined in the secondary readout from the primary screen, for Cb*Cln3*$^{+/+}$ ("+/+") DMSO-treated cells versus Cb*Cln3*$^{\Delta ex7/8/\Delta ex7/8}$ cells ("Δex7/8/Δex7/8"), treated either with DMSO or with the indicated compounds. Welch's t-test was used to determine statistical significance due to unequal sample size/variance in the DMSO-treated versus the compound-treated datasets. ****, P<0.0001; **, P<0.01; *, P<0.05

Figure S5.

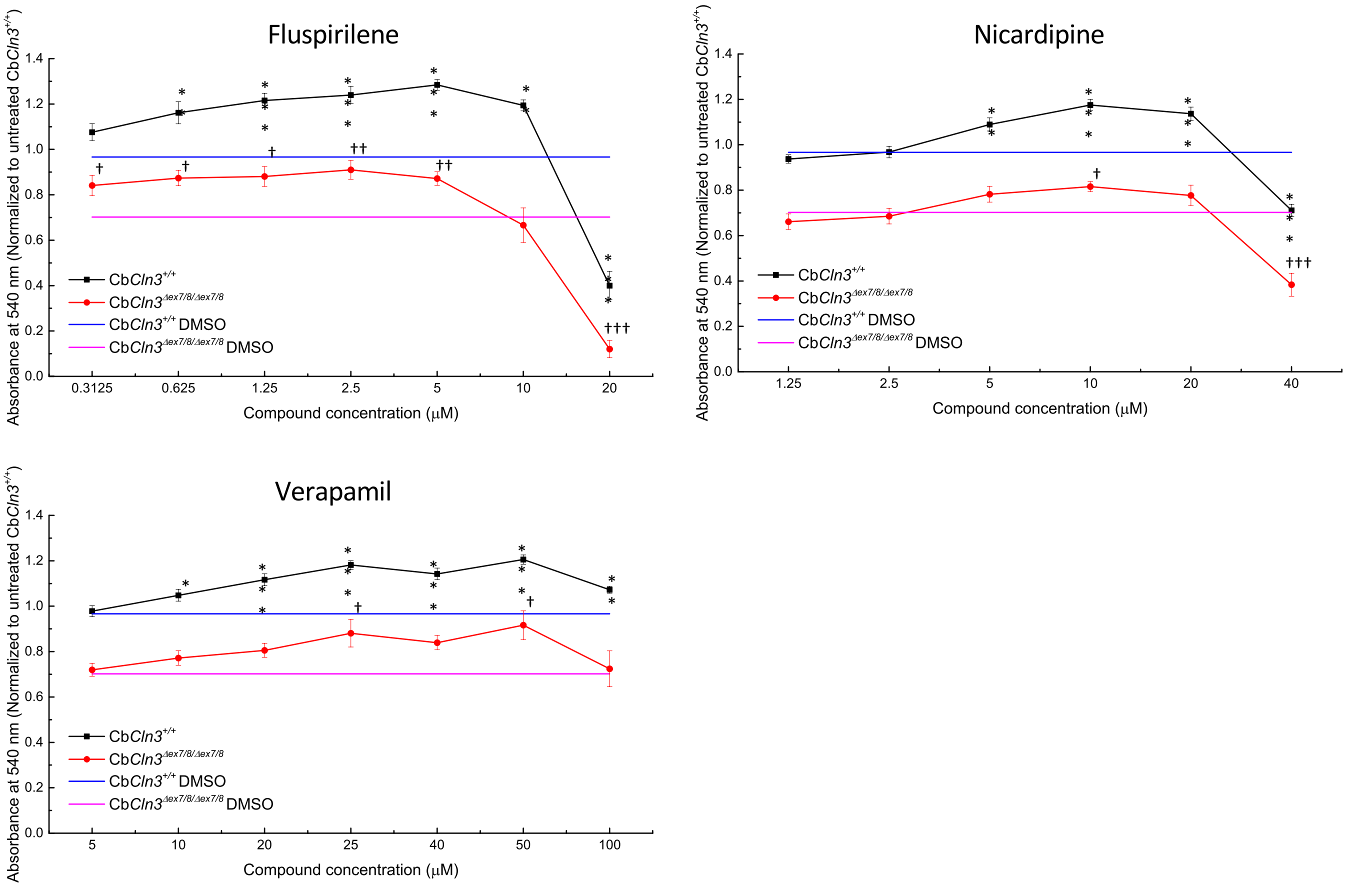

Figure S5. MTT assay toxicity analysis for fluspirilene, nicardipine, and verapamil. Graphs show relative toxicity as determined by MTT assay over the dose ranges indicated on the x-axis (compound treated Cb*Cln3*$^{+/+}$ cell values are shown in black, compound treated Cb*Cln3*$^{\Delta ex7/8 \Delta ex7/8}$ cell values are shown in red). Data were normalized to the untreated wild-type (Cb*Cln3*$^{+/+}$) cell values. Mean DMSO treated values are shown by the blue line (Cb*Cln3*$^{+/+}$ cells) and the pink line (Cb*Cln3*$^{\Delta ex7/8 \Delta ex7/8}$ cells). Data represent mean ± SEM of n=3 experiments. *, P < 0.05; **, P <0.01; ***, P < 0.001 of DMSO control Cb*Cln3*$^{+/+}$ cells tested against treated Cb*Cln3*$^{+/+}$ cells and †, P < 0.05; ††, P < 0.01; †††, P < 0.001 of DMSO control Cb*Cln3*$^{\Delta ex7/8/\Delta ex7/8}$ cells tested against treated Cb*Cln3*$^{\Delta ex7/8/\Delta ex7/8}$ cells (one-way ANOVA with Fisher post-hoc test).
